# A novel germline hyperactivating *JAK2* mutation L604F

**DOI:** 10.1007/s00277-023-05423-y

**Published:** 2023-08-28

**Authors:** Lukáš Dvořáček, Jana Marková, Aleš Holoubek, Dana Grebeňová, David Kundrát, Kateřina Kuželová, Jiří Schwarz

**Affiliations:** 1 Department of Proteomics, Institute of Hematology and Blood Transfusion, Prague, Czech Republic; 2 Clinical Department, Institute of Hematology and Blood Transfusion, Prague, Czech Republic; 3 Department of Genomics, Institute of Hematology and Blood Transfusion, Prague, Czech Republic

**Keywords:** Hereditary erythrocytosis, Polycythemia vera, Germline mutation, STAT5, *JAK2* F595

## Abstract

Somatic *JAK2* mutations are the main molecular cause of the vast majority of polycythemia vera (PV) cases. According to a recent structural model, the prevalent acquired V617F mutation improves the stability of the JAK2 dimer, thereby enhancing the constitutive JAK2 kinase activity. Germline *JAK2* mutations usually do not largely alter JAK2 signaling, although they may modulate the impact of V617F. We found an unusual germline *JAK2* mutation L604F in homozygous form in a young PV patient, along with a low allele burden *JAK2* V617F mutation, and in her apparently healthy sister. Their father with a PV-like disease had L604F in a heterozygous state, without V617F. The functional consequences of *JAK2* L604Fmutation were compared with those induced by V617F in two different in vitro model systems: (i) HEK293T cells were transfected with plasmids for exogenous JAK2-GFP expression, and (ii) endogenous *JAK2* modifications were introduced into HeLa cells using CRISPR/Cas9. Both mutations significantly increased JAK2 constitutive activity in transfected HEK293T cells. In the second model, *JAK2* modification resulted in reduced total JAK2 protein levels. An important difference was also detected: as described previously, the effect of V617F on JAK2 kinase activity was abrogated in the absence of the aromatic residue F595. In contrast, JAK2 hyperactivation by L604F was only partially inhibited by the F595 change to alanine. We propose that the L604F mutation increases the probability of spontaneous JAK2 dimer formation, which is physiologically mediated by F595. In addition, L604F may contribute to dimer stabilization similarly to V617F.

**Supplementary Information:**

The online version contains supplementary material available at 10.1007/s00277-023-05423-y.

## Introduction

*JAK2* tyrosine kinase somatic mutations are the most common genetic aberrations in myeloproliferative neoplasms (MPN). They are demonstrated in approximately 98% of patients with polycythemia vera (PV) and in 50 – 60% of patients with essential thrombocythemia and primary myelofibrosis [1]. Irrespective of the disease subtype, they are not only driver mutations but also confer a highly significant increase in thrombotic risk in patients with MPN.

The vast majority of the mutations occur in codon 617 in exon 14 (the V-F change) [1–5]. At the molecular level, the *JAK2* V617F point mutation increases ligand-independent JAK2 kinase activity. Rare alternative somatic mutations of *JAK2* have been described in exon 12 in PV [6, 7]. In addition, several dozen alternative somatic or germline “noncanonical” mutations of *JAK2* were found in rare MPN, MPN-like or hereditary polyglobulia or thrombocytosis cases.

JAK2 is a nonreceptor tyrosine kinase from the Janus kinase (JAK) family. JAK proteins are structured into several domains. The N-terminal part contains the receptor binding domains FERM and SH2. The tyrosine kinase (TK) domain is located at the C-terminus. TK domain is preceded by a pseudokinase (PK) domain, which is structurally similar to TK, but its catalytic activity is low. A new, two-step activation model has been recently proposed on the basis of structural analysis of a JAK1/dimeric receptor complex [8]. In the autoinhibited form, the TK domain is folded on the FERM-SH2 domain, hiding the activation loop and the kinase active site. This closed conformation also prevents JAK dimerization. Transient opening of the monomer is a prerequisite for JAK dimerization and full activation.

JAK hyperactivating mutations were proposed to act by two distinct mechanisms: destabilizing the monomeric closed state or stabilizing the dimeric open state [8]. Mutations of the first type are usually located at the interface between the TK domain and FERM-SH2/PK domains. These are often found in B-cell acute lymphoblastic leukemia [9]. In MPN, the most frequent example of the first mutation type is germline *JAK2* R1063H [10]. Mutations of the second type affect the interface between the two JAK monomer units, which are connected through their SH2-PK regions. Specifically in JAK2, the residues F537, F595, and V617 are at the core of this interface. According to the proposed model, the V617F change improves the compatibility of the interacting surfaces and thereby stabilizes the dimer [8]. Somatic mutations in exon 12 have similar effects as V617F [6, 7]. Importantly, the presence of an aromatic residue at position 595 is essential for dimer formation, and the F595 change to alanine abrogated the activating effect of V617F as well as of other *JAK2* mutations (K539L, R683G, T875N) [11].

While all of the clear somatic *JAK2* mutations in MPN lie in the PK domain, germline mutations may also be found in the kinase and FERM domains. Some germline mutations of *JAK2* may predispose patients to overt MPN [12, 13]. By themselves, these germline mutations usually do not induce substantial changes in JAK2 activity. However, they may augment the effect of V617F on JAK2 signaling in MPN, as described for the R1063H mutation [10, 14] or for the germline T108A [15]. Germline JAK2 mutations can be clinically silent or lead to slight elevations in blood counts (erythrocytes, platelets) or increments in thrombotic risk.

The diagnosis of overt MPN according to the World Health Organization (WHO) criteria [16] is relatively easy – parameters of the blood picture, histopathology, and molecular analyses are combined. However, in routine practice, many cases of polyglobulia and thrombocytosis lack either the typical MPN mutations in the *JAK2, CALR* or *MPL* genes, the histopathological features of MPN, or both. The underlying cause of these states is difficult to find – reactive changes or familial background may not be clearly demonstrated [17]. In recent years, next-generation sequencing (NGS) has often been used to elucidate the molecular background of these conditions [18–20].

## Material and methods

A detailed description of the methods is given in the Supplementary Information.

### The patient and family members

Peripheral blood samples were taken from the patient and her family members for subsequent molecular genetics studies. For practical reasons, buccal mucosa smears were used instead of the peripheral blood in the case of patient´s grandmother. DNA and cDNA were prepared using standard procedures. The patient´s diagnosis was made according to the CZEMP (Czech Group for Ph- Myeloproliferative Disorders) criteria [21].

### Diagnostic molecular tests

The presence of the *JAK2* V617F mutation in peripheral blood samples was tested by a real-time allelic discrimination method as described [22]. For NGS studies, an in-house custom panel to detect mutations in 37 genes was used. We tested the whole sequence of the given genes unless stated otherwise: *JAK2*, *CALR* (exon 9), *MPL*, *TET2*, *ASXL1* (exon 12), *DNMT3A*, *CBL* (exons 8, 9, 12), *TP53*, *AML1* (*RUNX1*) (exons 1–6), *EZH2*, *IKZF1*, *IDH1* (exon 4), *IDH2* (exon 4), *U2AF1*, *LNK* (*SH2B3*), *CUX1*, *NFE2* (exons 2–3), *KRAS* (exons 2–3), *NRAS* (exons 2–3), *SRSF2* (exons 1–2), *SF3B1* (exons 12–17), *GATA2* (exons 2–6), *PTPN11* (exons 2–15), *CSF3R* (exons 13–17), *SETBP1* (exon 4)*, WT1, ZRSR2, CEBPA, EPOR, EPO, VHL, HIF2α* (*EPAS1*)*, PHD2* (*EGLN1*)*, HBB, HBA1, HBA2, *and* BPGM.* The threshold of variant allele frequency (VAF) for NGS results was set to 5%. To detect the L604F mutation in family members not tested by NGS, direct Sanger sequencing using the Big Dye Terminator kit v. 3.1 (Applied Biosystems, Branchburg, NJ, USA) was performed. The same primers (IDT, Leuven, Belgium) were used for the real-time allelic discrimination of *JAK2* V617F [22] and for sequencing of the L604F variant.

### Preparation of *JAK2*-eGFP plasmids

Plasmids with *JAK2* wild-type (WT) or *JAK2* V617F were constructed by PCR-based techniques of molecular cloning by incorporating *JAK2* WT or *JAK2*-V617F sequences from pDONR223 plasmids containing respective genes [Addgene plasmids # 23,915 and # 81,756 [23, 24]] into plasmid pEGFP-N2 (originally Clontech, Mountain View, CA, USA) designed for exogenous expression of proteins with a green fluorescent protein (eGFP) tag.

In the second step, the *JAK2* L604F mutation was introduced into pEGFP-N2-JAK2 WT or pEGFP-N2-JAK2 V617F via site-directed mutagenesis (Q5 Site-Directed Mutagenesis Kit, New England Biolabs, Ipswich, MA, USA). To evaluate the importance of the aromatic residue F595, *JAK2* F595A was introduced into three pEGFP-N2-JAK2 plasmids with different mutations of *JAK2* (V617F, L604F, or V617F + L604F) via site-directed mutagenesis using the same kit.

All the mentioned plasmid constructs were checked by sequencing.

### Cell lines

HeLa and HEK293T cells were obtained as a gift from Dr. Š. Němečková (Institute of Hematology and Blood Transfusion, Prague, Czech Republic) and authenticated using analysis of short tandem repeats. The results were compared with the ATCC database.

### Cell transfection

The plasmids with different *JAK2* variants were transfected into HEK293T cells using jetPRIME transfection reagent (Polyplus Transfection, Illkirch, France) following the manufacturer’s instructions. The cells were cultured for 24 h and harvested for western blot analysis. An aliquot of each sample was used to determine the transfection efficiency using a BD Fortessa flow cytometer (Becton–Dickinson, Prague, Czechia).

### Western blot

Protein amounts in cells with exogenous or endogenous JAK2 variants were assessed using standard protocols for western blotting. The antibodies used were the following: JAK2 (Cell Signaling, #74987S), phospho-JAK2 Tyr1007/1008 (Cell Signaling, #3771S), STAT5 (Cell Signaling, #25656), pSTAT5 Tyr694 (Cell Signaling, #9359), STAT3 (Cell Signaling, #4904), pSTAT3 Tyr705 (Cell Signaling, #9145), and β-ACTIN (Santa Cruz, sc-47778).

### *JAK2* modification by CRISPR

V617F or L604F *JAK2* mutations were introduced using the CRISPR/Cas9 technique. HeLa cells were transfected using nucleofection (4D-Nucleofector X Unit, Lonza, Basel, Switzerland), seeded in 6-well plates and cultured for 3 days. To obtain stable clones with *JAK2* mutations, HeLa cell suspensions were diluted to 5 cells/ml, and 100 µl aliquots were distributed into a 96-well culture plate. Cell growth was regularly monitored by visual inspection, and wells containing a single colony were selected for further cell expansion and analysis. *JAK2* mutation status was then checked by sequencing.

## Results

### The patient and her family

An 18-year-old female was referred from another hospital to the Institute of Hematology and Blood Transfusion (IHBT) in Prague on November 27, 2020, because of accidental findings of polyglobulia and thrombocytosis. She had a history of mild palpable splenomegaly in childhood, which spontaneously resumed. Her pediatrician has never indicated a hematological examination. She had two by chance examinations of the blood counts within 2013–2018, showing consistently elevated platelet counts (528–621 × 10^9^/L, lately also with elevated parameters of the red cell lineage – Hct 0.51, RBC 6.08 × 10^12^/L). On November 12, 2020, when checked in another Prague hospital, her blood count was: WBC 8.9 × 10^9^/L, RBC 7.95 × 10^12^/L, Hb 176 g/L, Hct 0.589, and Plt 449 × 10^9^/L. Four phlebotomies were performed. Nevertheless, her erythropoietin level was low (0.9 U/L). She was asymptomatic, without previous thrombotic events. Her spleen was not palpable but was enlarged by sonography (18 cm in the long axis). In addition, a 2 cm accessory spleen was noted. At IHBT, real-time allelic discrimination revealed a heterozygous *JAK2* V617F mutation with 21% mutant allele burden. The diagnosis of PV was made according to the CZEMP recommendations [21]. She received treatment with low-dose acetylsalicylic acid and her last (5^th^) phlebotomy was performed, leading to control of her hematocrit level (< 0.45), yet with persistence of increased platelet counts. Therefore, therapy with anagrelide (ANG; Thromboreductin®) was commenced, leading to platelet count normalization (< 400 × 10^9^/L). The patient had borderline liver function tests, probably due to antibodies to hepatitis viruses both A and B. She had autoimmune thyroiditis with a high level of anti-thyroid tissue antibody, along with a thyroid nodule. For these reasons, the treatment of choice, pegylated interferon-α (IFN; Pegasys®), was administered only later, in November 2022. As of March 2023, she received 90 µg IFN weekly and 0.5 mg ANG daily. Her blood counts are within normal limits.

**Fig. 1:**
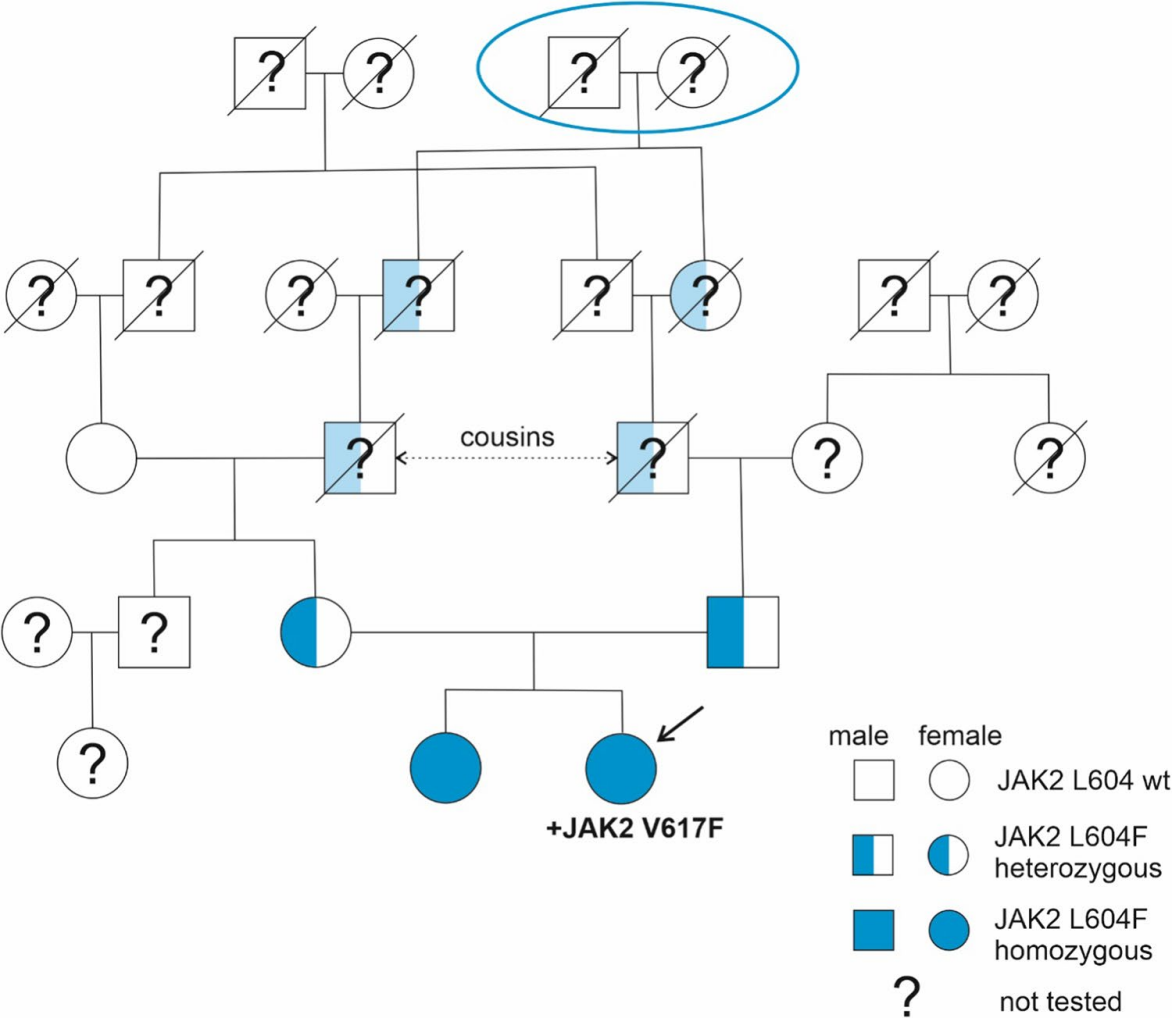
The pedigree of the family with the *JAK2* L604F mutation. The patient and her sister have the L604F mutation (blue) in a homozygous state, and her parents have heterozygous L604F. The germline L604F probably arises from a common ancestor pair (encircled in blue). The presence of the mutation in the nontested family members is presumed (light blue) as a highly likely way to yield the results obtained in the tested members

NGS revealed no other somatic mutation except *JAK2* V617F. However, it also showed a previously undescribed *JAK2* L604F mutation with a surprising VAF of 99.5%, which suggested that it had been inherited from both parents. We screened her family (sister, parents and maternal grandmother) for the presence of the L604F mutation and found it in a heterozygous state in both parents, and homozygous in her older sister. The result of her grandmother was negative. The possible presence of *JAK2* V617F in the family members was also tested by the real-time allelic discrimination method (1% sensitivity), and the results were negative, except for the patient. Taking a thorough family history, we found that the patient´s ancestors came from two small nearby villages in South Bohemia and that the patient´s grandfathers were cousins (Fig. 1). Similar to the patient herself, her older sister had mild splenomegaly after birth, which is currently not palpable. She had a normal blood count except for a slightly higher WBC count (11.5 × 10^9^/L). The patient´s mother had a normal blood count, and her Hct (0.450) was above average, perhaps due to mild asthma. The father´s blood counts strongly suggested a PV diagnosis (WBC 13.7 × 10^9^/L, Hct 0.512, Plt 599 × 10^9^/L). He had no other mutation in addition to *JAK2* L604F, as detected by the NGS panel. He refused all further examinations (including trephine biopsy), as well as treatment and follow-up.

### Experimental procedures

The functional consequences of the L604F *JAK2* mutation were analyzed in two different model systems and compared with those of V617F.

#### Increased kinase activity of mutated JAK2 variants produced from plasmids in HEK293T cells

**Fig. 2:**
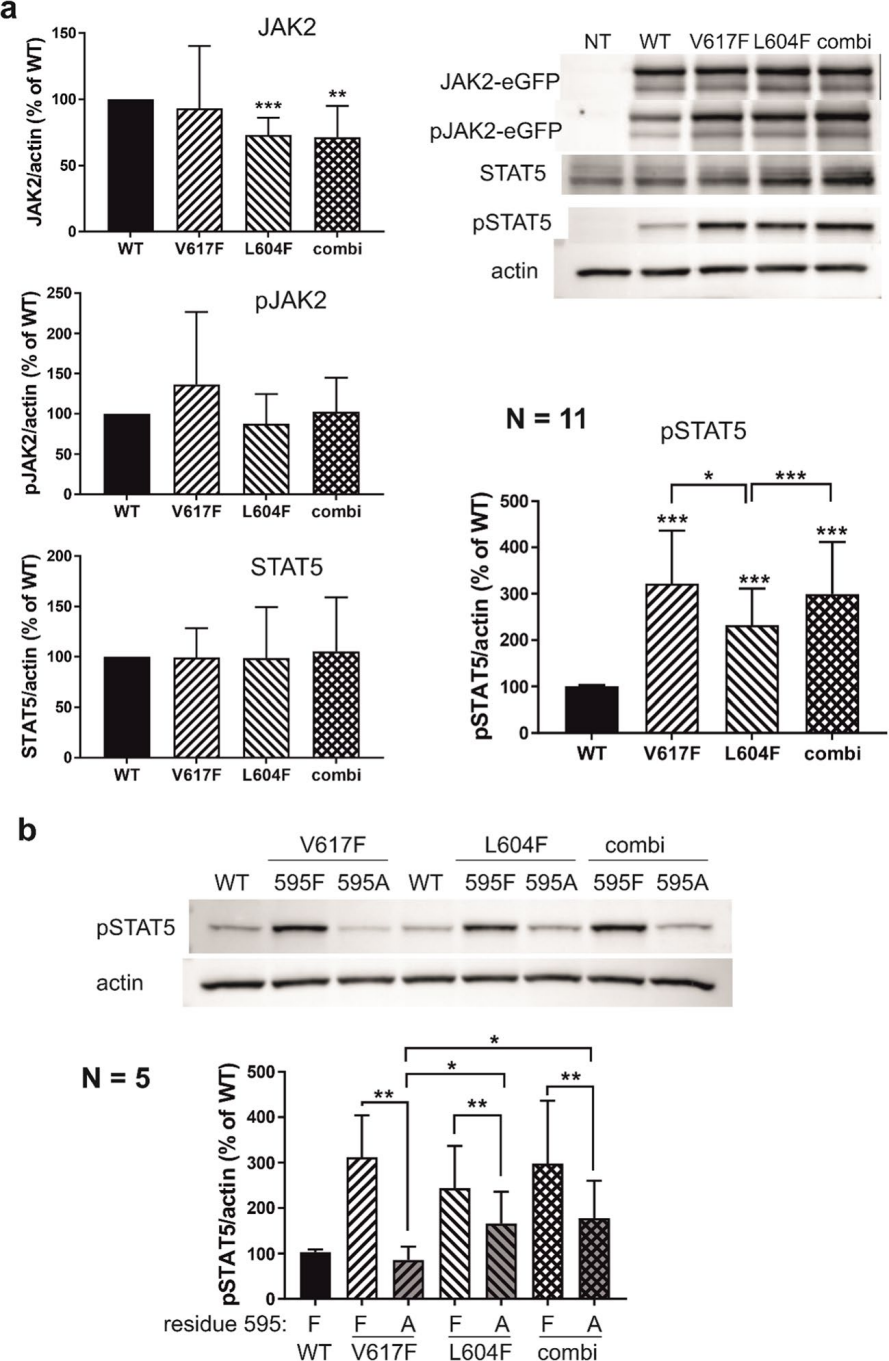
Effect of *JAK2* mutations in HEK293T cells with exogenous *JAK2* variants. **a**. Western blot analysis of HEK293T cells transfected with plasmids encoding different *JAK2* variants (WT, V617F, L604F, and the V617F + L604F combination) fused with eGFP. Top right: representative images of JAK2 and STAT5 protein amounts and phosphorylation status of JAK2 Tyr1007/1008 and STAT5 Tyr694. ACTIN was used as the loading control. NT – untransfected cells. The bar plots show relative band intensities from repeated experiments (mean ± SD, N = 11). The band intensities were related to those of the WT sample (black bars, 100%) and normalized using ACTIN amounts and eGFP-positive cell fraction as determined by flow cytometry. Paired Student´s t test was used to evaluate differences between each mutated form and WT (*** *p* < 0.001) as well as between L604F and V617F (* *p* < 0.05) or between L604F and the L604F + V617F combination (*** *p* < 0.001). **b**. Effect of *JAK2* F595A mutation on STAT5 hyperphosphorylation induced by the mutations V617F, L604F, or their combination. Top: representative images of western-blot membranes. Bottom: means ± SD of relative band intensities for STAT5 Tyr694 antibody from 5 transfection experiments. Data processing was the same as in a. Paired Student´s t test was used to evaluate differences between different mutated forms (* *p* < 0.05, ** *p* < 0.01) as indicated

We prepared a system for exogenous expression of JAK2 fused with a green fluorescent protein (eGFP). The tag allowed for checking the protein production using flow cytometry as well as for separation of the exogenous and endogenous JAK2 forms in western blots (WB). HEK293T cells were transiently transfected with plasmids encoding wild-type *JAK2* (WT), *JAK2* with the V617F mutation (*JAK2* V617F), *JAK2* with the L604F mutation (*JAK2* L604F), and *JAK2* with the V617F + L604F combination (*JAK2* combi). After 24 h of culture, the cells were harvested, aliquots were used to check the transfection efficiency, and the remaining cells were lysed and used for protein analysis by WB. The phosphorylation sites JAK2 Tyr1007/1008 and STAT5 Tyr694 were used as indicators of JAK2 kinase activity. The intensity of the WB bands was normalized using ACTIN amounts. The transfection efficiency varied between 18 and 64%, independent of the mutation type, and WB results for pSTAT5, pJAK2, and JAK2 were corrected using eGFP-positive cell fraction values. Figure 2(panel a) shows representative WB membranes and a summary of the results from repeated experiments. The amounts of eGFP-labeled JAK2 were slightly lower in samples transfected with L604F or combi *JAK2* variants than in those transfected with the WT form. The signal of the phospho-specific JAK2 antibody (Tyr1007/1008) was comparable in all the samples. In contrast, the downstream target of JAK2 (STAT5) was clearly more phosphorylated by all the mutated variants compared to JAK2 WT. In the untransfected control (NT), STAT5 phosphorylation at Tyr694 was undetectable. JAK2 overexpression resulted in a marked increase in pSTAT5 band intensity for all the tested JAK2 variants. All the mutated forms had a significantly higher effect than the wild-type form (*p* < 0.001 for all comparisons), although the increase induced by the L604F mutation had a lower amplitude than that induced by V617F. The combination of L604F with V617F did not enhance the impact of V617F alone.

#### Analysis of the importance of F595 for increased JAK2 activity

The aromatic residue F595 is required for enhanced ligand-independent kinase activity of several mutated JAK2 variants, including JAK2 V617F [11, 25]. Therefore, we tested the effect of the F595A mutation on the activity of JAK2 with V617F, L604F, and the V617F + L604F combination. As shown in Fig. 2b, introduction of the F595A mutation completely abolished the increase in STAT5 phosphorylation induced by V617F. In contrast, the addition of F595A to L604F or combi variants had only a partial inhibitory effect on JAK2 hyperactivation. In fact, the L604F mutation increased JAK2 kinase activity even in the absence of an aromatic residue at position 595. Closely similar results were also obtained for another JAK2 downstream target, STAT3, using Tyr705 as the marker phosphorylation site (Supplementary Information, Figure S1). The amplitude of changes in STAT3 phosphorylation was lower than for STAT5, and the pTyr705 levels were already detectable in untransfected cells, but the main trends were the same as for STAT5.

#### Impact of endogenous JAK2 mutations in HeLa cells

**Fig. 3:**
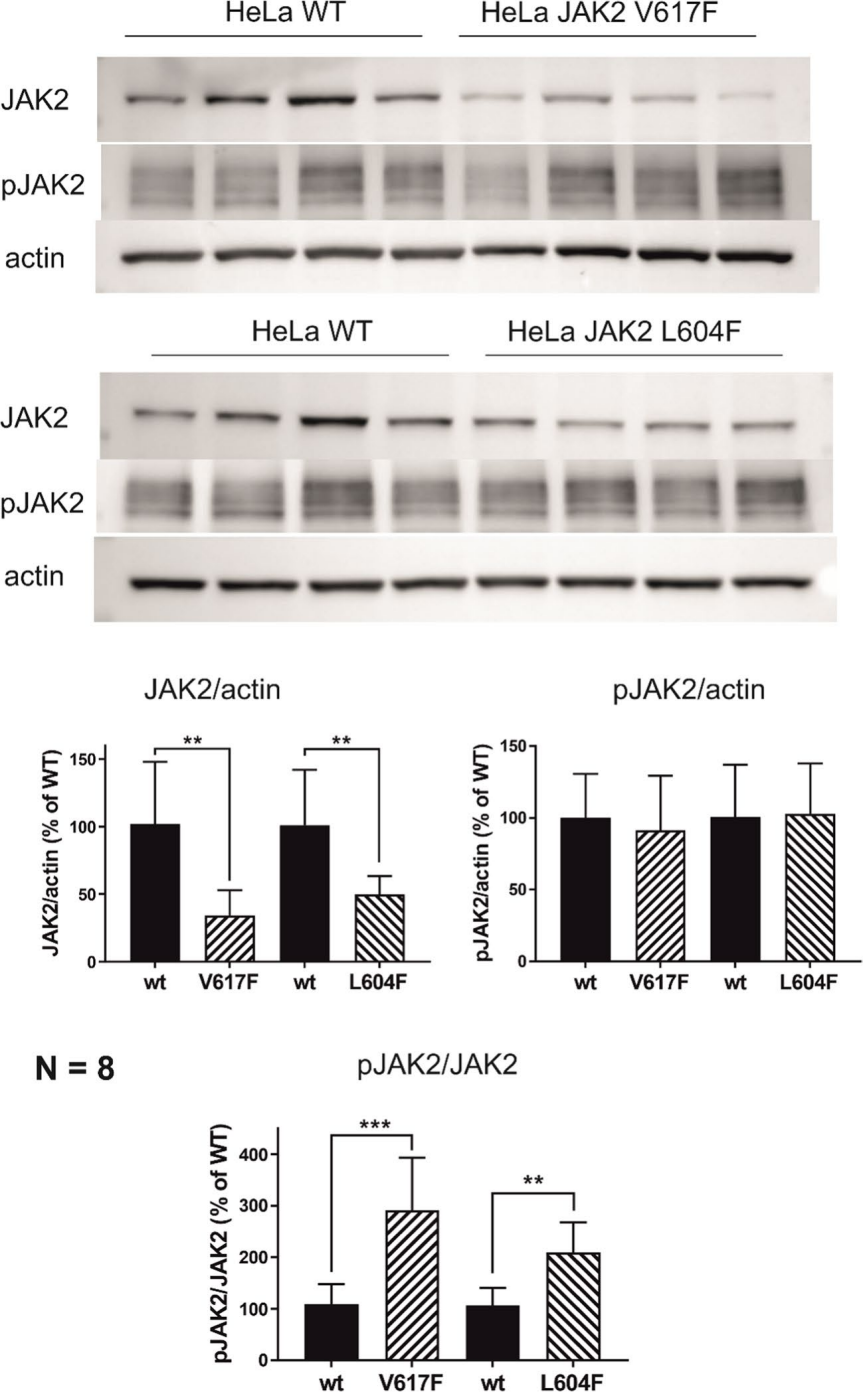
Effect of endogenous *JAK2* mutations in HeLa cells. Western blot analysis of HeLa cells with V617F or L604F *JAK2* mutations introduced by CRISPR. The expected gene modification was confirmed by sequencing. Eight independent harvests were performed for each modified subline and the wild type (WT) parental line. The cell lysates were analyzed in groups – each western-blot membrane contained 4 WT samples and 4 samples from one mutated subline. The measured band intensities were normalized to ACTIN and related to the mean value from WT samples included in the given membrane (100%). Top: representative examples of JAK2 and pJAK2 Tyr1007/1008 signals. ACTIN was used as the loading control. Bottom: means ± SD of the relative band intensities from 8 independent samples for each *JAK2* variant. The differences between modified sublines and the WT control were evaluated by unpaired Student´s t test (** *p* < 0.01, *** *p* < 0.001)

Subsequently, we introduced the V617F or L604F mutation using the CRISPR gene modification method into the genome of HeLa cells. Again, we compared the amounts and phosphorylation status of JAK2 and STAT5. *JAK2* sequencing confirmed the presence of the desired modification in the obtained cell clones, in parallel with a wild-type allele. The impact of *JAK2* mutations in this system was different from that observed for exogenously produced JAK2. Compared to the parental wild-type population, the total amount of JAK2 was markedly reduced in the clones with *JAK2* activating mutations, whereas the amount of the phosphorylated (kinase active) form was similar (Fig. 3). Consistently, we did not observe any change in STAT5 phosphorylation (not shown). The extent of JAK2 autophosphorylation represented by the pJAK2/JAK2 ratio increased in the clones with mutated *JAK2* compared to the parental cells (Fig. 3, bottom). The effect of L604F tended to be lower than that of V617F (*p* = 0.07).

## Discussion

From the clinical point of view, the current report shows two young sisters with a previously undetected homozygous germline mutation, *JAK2* L604F. Both sisters had palpable splenomegaly at birth, which resumed spontaneously. It may be speculated that after birth, the spleen was palpable as the remnant of the hepatolienal period of fetal hematopoiesis; after birth, bone marrow takes over the production of blood elements. One of the sisters (the patient) developed overt PV with acquisition of the V617F mutation, while the other remained symptomless.

The germline mutations described to date usually have limited impact on JAK2 kinase activity [10, 15, 26–29]. A combination of two inherited heterozygous *JAK2* mutations (R1063H and E846D), each of them only slightly activating, was found in a patient with erythrocytosis and megakaryocytic atypia [10]. In contrast, the germline mutation L604F was clearly hyperactivating, although the amplitude of changes was somewhat lower compared with those induced by the prominent somatic change V617F (Fig. 2). It thus seems that the L604F mutation might also explain the PV-like blood picture of the patient´s father. However, if so, the family history (Fig. 1) incites questions: 1) why is the impact of the germline mutation so different in two L604F heterozygotes? The patient´s mother has normal blood counts, while her father´s blood counts are highly reminiscent of full-blown PV. 2) Analogically, why does the heterozygous father have “more disease” than the patient´s homozygous sister? Various genetic predisposition conditions, such as the *JAK2* haplotype, or many other factors may influence the probability of developing an MPN phenotype following acquisition of a somatic JAK2 mutation [12, 30]. However, these factors are thought to impact mainly the rate of expansion of the mutated clone [30] and it is unclear if they are also relevant for germline mutations. Some yet unknown or known processes of aging and immunity may also be involved [30, 31]. Our report is in line with the notion that MPN presenting at a young age may develop on a germline mutation background, perhaps needing a low allele load driver mutation for a complete disease phenotype [12]. A remark goes to the issue of “negative family history”. Our case illustrates that a hereditary disease or predisposition cannot be excluded on this basis. To be uncovered, germline changes need to produce some symptoms, and even the true disease may be symptomless and/or unrecognized.

Recent structural analysis of JAK1 associated with an engineered dimeric cytokine receptor has highlighted the importance of the PK domain in JAK1 dimerization, which is a prerequisite for JAK kinase activity [8]. According to this model, which is very likely also valid for JAK2, F595 would be in the core of the interface between the two interacting PK domains in a JAK2 dimer. Several hyperactivating *JAK2* mutations, including V617F, improve the compatibility of the two interacting surfaces and enable cytokine-independent dimerization. However, the constitutive activity of these mutants strongly depends on F595 [11]. The novel mutation L604F introduces an aromatic residue near F595, and the F-A change in codon 595 also reduced the kinase activity of JAK2 with L604F (Fig. 2b), which confirms the importance of F595. However, whereas the F595A mutation completely abrogated the hyperactivating effect of V617F, higher signaling activity was partially maintained after F595A introduction to *JAK2* L604F. With regard to the proximity of F604 to F595, we suggest that the L604F mutation might partly compensate for the loss of F595, helping JAK2 dimer formation. Thus, L604F could promote spontaneous dimer formation in cooperation with F595. In addition, L604F could contribute to dimer stability similarly to V617F, albeit somewhat less efficiently.

The mechanisms causing clinical symptoms of MPN induced by *JAK2* V617F have not been completely elucidated. JAK/STAT signaling is clearly involved but JAK2 has many different functions independent of STAT activation. The mutant JAK2 variant enters the cell nucleus [32] and promotes epigenetic modifications, for example through phosphorylation of the histone H3 [33] and the protein arginine methyltransferase PRMT5 [34, 35]. Heritable JAK2 mutations described to date did not induce significant changes of JAK2 kinase activity in cell line models, and hyperactivation of JAK/STAT signaling is considered the likely reason of the fact that the V617F mutation is incompatible with survival during embryonic development. However, the germinal L604F mutation described in this work is clearly associated with increased STAT5 phosphorylation (Fig. 2). This indicates that embryonic lethality due to V617F might be related to STAT5-independent JAK2 functions, which could remain unchanged in JAK2 L604F. Such functions might also contribute to variable clinical symptoms of MPN driven by the JAK2 V617F mutation.

The observed effects of *JAK2* mutations varied in different model systems. Forced JAK2 overproduction in transfected HEK293T cells resulted in increased JAK2 signaling reflected by higher phosphorylation at STAT5 Tyr694 (Fig. 2a) and STAT3 Tyr705 (Supplementary Figure S1). In the second model system, the amounts of pJAK2 and pSTAT5 in the clones with mutant JAK2 were the same as in the parental cells, whereas the total JAK2 levels were markedly lower (Fig. 3). This suggests that the mutations might limit JAK2 protein production or stability. Indeed, JAK2 levels are regulated through proteasome-mediated degradation, and the V617F mutation is associated with a higher degradation rate [36]. Consistently, lower amounts of JAK2 with V617F in comparison with the wild-type form were previously observed in HEK293T cells transfected with *JAK2* mutants, despite higher levels of phosphorylated (active) JAK2 [14]. Altogether, these findings indicate that *JAK2* mutation may not have a large impact on protein signaling in cells with regulated total JAK2 levels. Nevertheless, a reduced overall amount of JAK2 might predispose such cells to the cumulation of compensatory mutations. Thus, the germline L604F mutation may result in lower JAK2 levels and promote preferential expansion of cells that acquire the additional V617F mutation. Interestingly, the activating mutations only slightly altered the total JAK2 levels in our first model system, where large amounts of exogenous JAK2 were produced in HEK293T cells (Fig. 2a). In this case, the addition of the eGFP label to JAK2 might have interfered with protein degradation. Alternatively, a negative feedback loop might limit JAK2 production in cells with high pJAK2 levels. In this case, exogenous protein production could be out of control by such regulatory elements.

## Conclusions

We describe a novel hyperactivating *JAK2* mutation, L604F, found in a patient with PV and her family. In our in vitro models, the impact of the germline L604F mutation approached that of the common somatic driver mutation V617F, in line with the presumed localization of both mutations at the interface between JAK2 monomers in the kinase-active dimer. Consistent with the recently published structure analysis [8], we propose that the introduction of an aromatic residue at the 604 position could facilitate spontaneous JAK2 dimer formation and/or contribute to dimer stability. The germline mutation JAK2 L604F is the likely cause of splenomegaly at birth. Furthermore, this mutation might explain the PV-like phenotype in the patient´s father with heterozygous *JAK2* L604F, who had none of the currently known causal mutations. However, the presence of the *JAK2* L604F mutation in itself is not sufficient to induce overt disease, as other members of the family were symptomless.

The L604F mutation could be demonstrated thanks to our NGS panel allowing sequencing of the whole *JAK2* (as well as *MPL*) gene. We advocate this diagnostic approach not only in “triple negative” MPN patients (lacking driver mutations in *JAK2, CALR* or *MPL* as screened by the routine methods), but also in young individuals with MPN or unexplained polyglobulia or thrombocytosis, even in those with a low allele load of the known driver mutations. The “noncanonical” mutations may have prognostic and therapeutic consequences [18–20]. However, longer follow-up of patients with germline mutations is needed to answer issues about their real biological, clinical and prognostic relevance and about the optimal preventive measures and management in affected individuals.

## Supplementary Information

Below is the link to the electronic supplementary material.

Supplementary file1 (PDF 1296 KB)

## Data Availability

All data generated or analyzed during this study are included in this published article and its supplementary information files. The described material (plasmids, genetically modified cells) is available from the corresponding author on reasonable request.
