# Supplementary material for: A novel germline hyperactivating JAK2 mutation L604F
Source: Ann Hematol. 2023 Aug 28;102(10):2725–34. doi: 10.1007/s00277-023-05423-y (PMC10492870; doi:10.1007/s00277-023-05423-y)

## Supplementary Information

## Annals of Hematology

### A novel germline hyperactivating *JAK2* mutation L604F

Lukáš Dvořáček, Jana Marková, Aleš Holoubek, Dana Grebeňová, David Kundrát, Kateřina Kuželová*, Jiří Schwarz

* Kateřina Kuželová, Institute of Hematology and Blood Transfusion, Prague, Czech Republic, kuzelova@uhkt.cz

## File content

Material and Methods - detailed description

Supplementary Figure S1

## Material and methods

### Next generation sequencing

The sequencing library was prepared using the SureSelect XT Library Preparation Kit and custom designed probes panel (Agilent Technologies, Santa Clara, CA, USA), targeting the genes chosen for the study. Libraries were sequenced on MiSeq (Illumina, San Diego, CA, USA) using the MiSeq Reagent Kit V2 for 300 cycles (Illumina). Sequence Pilot software (JSI medical systems, Ettenheim, Germany) and an in-house pipeline were used for analysis of the output data. Trimmed and filtered reads were mapped to GRCh38 genome and variants were selected for further analysis if they met the following criteria: minimal coverage of 500x, Phred score greater than 35, and variant allele frequency (VAF) of ≥ 0.05. Variants were analysed using Varsome, 1000 Genomes, dbSNP and other databases.

### Preparation of JAK2-eGFP plasmids

Plasmids with *JAK2* wild-type (WT) or *JAK2* V617F, both fused with eGFP, were constructed by PCR-based techniques of molecular cloning (extended primers, see Table 1A) by incorporating JAK2 WT or JAK2-V617F sequences from pDONR223 plasmids containing respective genes (Addgene plasmids # 23915 and # 81756 (28,29)) into plasmid pEGFP-N2 (originally Clontech, Mountain View, CA, USA) designed for exogenous expression of proteins with a green fluorescent protein (eGFP) tag, using SacI and XmaI restriction sites. pDONR223-JAK2 was a gift from William Hahn & David Root (Addgene plasmid # 23915 ; http://n2t.net/addgene:23915 ; RRID:Addgene_23915). pDONR223_JAK2_WT was a gift from Jesse Boehm & William Hahn & David Root (Addgene plasmid # 81756 ; http://n2t.net/addgene:81756 ; RRID:Addgene_81756).
In the second step, *JAK2* L604F mutation was introduced into pEGFP-N2-JAK2 WT or pEGFP-N2-JAK2 V617F via site-directed mutagenesis (Q5 Site-Directed Mutagenesis Kit, New England Biolabs, Ipswich, MA, USA). To evaluate the importance of the residue F595, *JAK2* F595A was introduced into three pEGFP-N2-JAK2 plasmids, each with different mutation of *JAK2* (V617F, L604F, or V617F + L604F) via site-directed mutagenesis using the same kit. Primers used are listed in Table 1A.

Table 1: Oligonucleotides used for *JAK2* cloning and modifications

A: Primers used for cloning and site-directed mutagenesis of *JAK2*-eGFP plasmids

| | Forward | Reverse |
|---|---|---|
| extended primers | AAAAAAGAGCTCAAATGGGAATGGCCTGCCTTACGAT | AAAAAACCCGGGTCCAGCCATGTTATCCCTTA |
| *JAK2* L604F | TTTTCTCACAAGCATTTGGTTTTAAATTATG | CTTGCTCATCATACTTGCTGCTTC |
| *JAK2* F595A | AGAGTCTTTCGCTGAAGCAGCAAG | GAATAGTTTCTGTGTGCTTTATC |

B: Guide RNAs and HDR templates used for *JAK2* modification by CRISPR

| | gRNA | HDR template |
|---|---|---|
| *JAK2* V617F | sgRNA: AATTATGGAGTATGTGTCTG | CAAGCTTTCTCACAAGCATTTGGTTTTAAATTATGGAGTATGTTTCTGTGGAGACGAGAGTAAGTAAAACTACAGGCTTTCTAATGCCTTTCTC |
| *JAK2* L604F | crRNA: AAGCAGCAAGTATGATGAGCA | TCCTTAGTCTTTCTTTGAAGCAGCAAGTATGATGAGCAAGTTTTCTCACAAGCATTTGGTTTTAAATTATGGAGTATGTGTCTGTGGAGACGA |

**Western-blot**

Cells were washed once with ice-cold phosphate buffered saline (PBS, pH 7.4) and scrapped into modified RIPA buffer (50 mM HEPES, 0.15 M NaCl, 2 mM EDTA, 0.1 % NP40, 0.05 % sodium deoxycholate, pH 7.2) with freshly added protease and phosphatase inhibitors (P8340, resp. P5726, Sigma-Aldrich). The suspension was then transferred to a centrifugation tube and incubated for 10 min at 4 °C. Cellular debris was removed by centrifugation (15 000 g/4 °C/15 min), the lysate was mixed 1:1 (v/v) with 2x Laemmli sample buffer and incubated for 5 min at 95 °C.

An equivalent of 20 µg of total protein was resolved in 7.5 % polyacrylamide gel and transferred to a nitrocellulose membrane. The membrane was blocked in PBS with 3 % bovine serum albumin for 1 h at room temperature and incubated with the primary antibody in TBS (20 mM Tris, 137 mM NaCl, pH 7.6) with 0.1 % Tween-20 (TBST) at 4 °C overnight. It was then washed six times in TBST and incubated with the corresponding HRP-conjugated secondary antibody for 1 h at room temperature. Chemiluminiscence signal from Clarity Western ECL Substrate (BioRad, #170 5060) was detected and analyzed using G:BOX iChemi XT-4 (Syngene, Cambridge, UK).

***JAK2* modification by CRISPR**

Guide RNAs (gRNA) and templates used to generate cells with *JAK2* mutations via homology-directed repair (HDR) are specified in Table 1B. Cas9 enzyme, Cpf1 enzyme, gRNA, and HDR templates were obtained from IDT (Integrated DNA Technologies, Coralville, IA, USA): Alt-R S.p. HiFi Cas9 Nuclease V3, Alt-R L.b. Cas12a (Cpf1) Ultra, Alt-R CRISPR–Cas9 sgRNA, Alt-R L.b. Cas12a crRNA, Alt-R HDR Donor Oligo. HeLa cells were transfected using nucleofection (4D-Nucleofector X Unit, Lonza, Basel, Switzerland). The transfection reaction contained $4 \times 10^5$ HeLa cells. For V617F mutation (*JAK2* V617F), 425 pmol Cas9 enzyme, 480 pmol sgRNA, and 500 pmol HDR template was used.
For L604F mutation (*JAK2* L604F), 504 pmol Cpf1 enzyme, 640 pmol crRNA, and 500 pmol HDR template was used. After transfection, HeLa cells were seeded in 6-well plates and cultured for 3 days. To obtain stable clones with *JAK2* mutations, Hela cell suspension was diluted to 5 cells/ml, and 100 µl aliquots were distributed into a 96-well culture plate. Cell growth was regularly monitored by visual inspection, and wells containing a single colony were selected for further cell expansion and analysis. *JAK2* mutation status was then checked by sequencing.

## Figure S1: Effect of *JAK2* mutations on STAT3 phosphorylation.

HEK293T cells were transfected with plasmids coding for different JAK2 variants fused with the green fluorescent protein, incubated for 24h and harvested into lysis buffer. Amount of STAT3 phosphorylated at Tyr705 was analyzed by western blot. The figure shows representative membranes (top) and relative band intensities from 5 repeated experiments (bottom). NT - untransfected control. The measured pSTAT3 band intensities were normalized using actin amounts and eGFP-positive cell fraction determined by flow cytometry. The value from NT sample was subtracted as the background. Samples transfected with mutated JAK2 variants (V617F, L604F, or the combination V617F + L604F = combi, with or without F595 change to alanin) were related to the sample transfected with wild-type JAK2 (WT, 100 %).

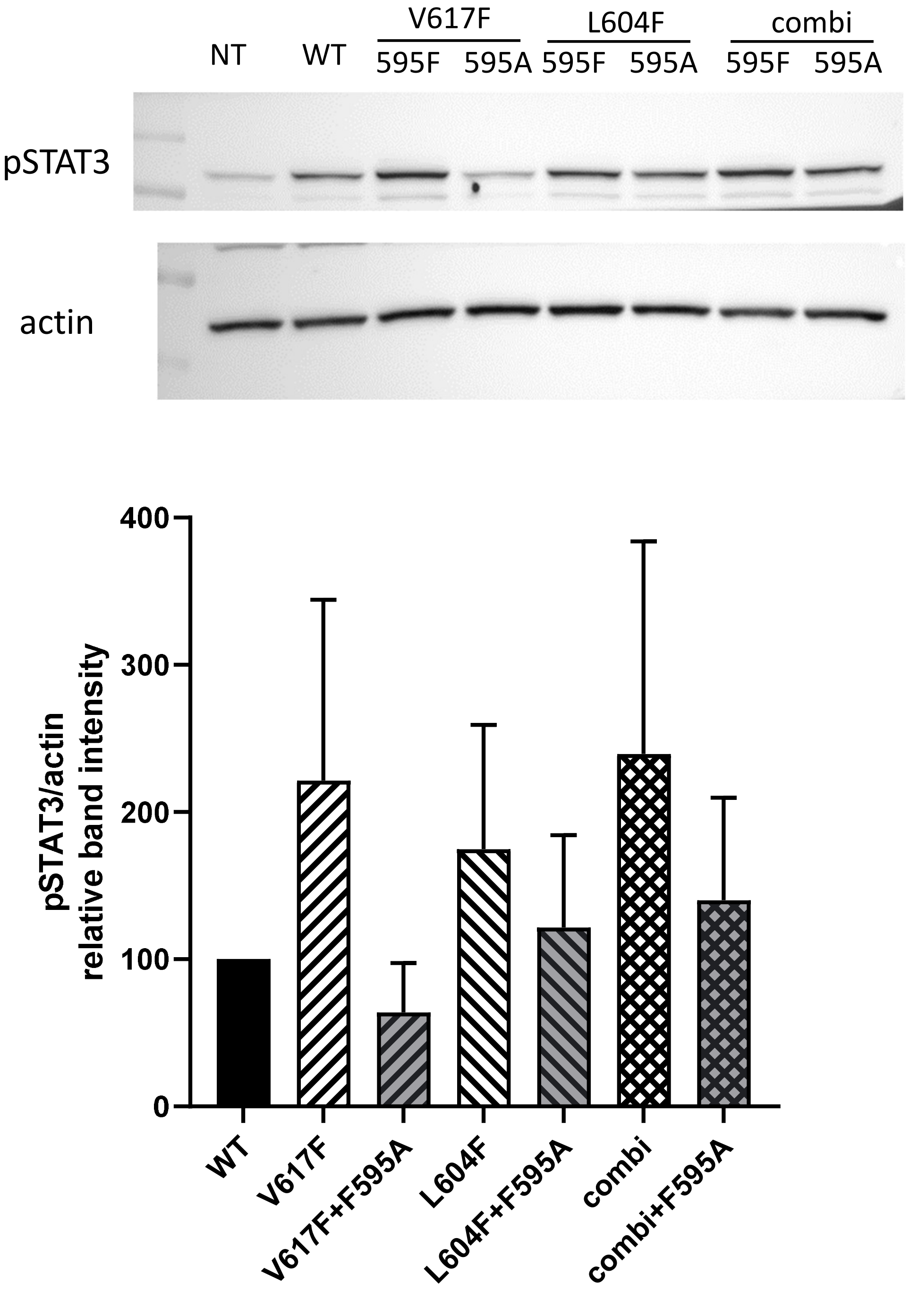

Supplement: Supplementary file 1 — Supplementary file1 (PDF 1296 KB) [file 277_2023_5423_MOESM1_ESM.pdf]
